# Patient satisfaction with prehospital emergency care following a hip fracture: a prospective questionnaire-based study

**DOI:** 10.1186/s12912-018-0307-x

**Published:** 2018-08-16

**Authors:** Glenn Larsson, Ulf Strömberg, Cecilia Rogmark, Anna Nilsdotter

**Affiliations:** 1 Department of Ambulance and Prehospital Care, Region Halland, Health Centre Nyhem, 302 49 Halmstad, Sweden; 2 Department of Orthopaedics, Lund University, Lund, Sweden; 3 Skane University Hospital, Malmö, Sweden; 4 Department of R&D, Sahlgrenska University Hospital, Göteborg, Sweden

**Keywords:** Prehospital emergency care, Patient satisfaction, Hip fracture, Ambulance nurse

## Abstract

**Background:**

Older patients with a hip fracture require specialized emergency care and their first healthcare encounter before arriving at the hospital is often with the ambulance service. Since 2005 there has been a registered nurse on the crew of every ambulance in Sweden in order to provide prehospital emergency care and to prepare the patients for hospitalization. It is important to investigate patient satisfaction with prehospital emergency care following a hip fracture to ensure that their expectations of good care are met.

The aim of this study was to investigate patient satisfaction with prehospital emergency care following a hip fracture by comparing two similar emergency care contexts.

**Methods:**

The study was conducted using the Consumer Emergency Care Satisfaction Scale (CECSS) on patients treated for hip fracture in prehospital emergency care. The data were collected within a randomized controlled study for the purpose of comparing prehospital fast track care (PFTC) and the traditional type of transport to an accident and emergency department (A&E).

**Results:**

Questionnaire data from 287 patients, 188 women (66%) and 99 men (34%) with a mean age of 80.9 years, were analysed. More than 80% of the patients selected the most positive response alternatives, but 16% were dissatisfied with the nursing information provided. Patients in PFTC responded more positively on specific caring behaviour than those transported to the A&E department in the traditional way.

**Conclusion:**

Patient satisfaction with prehospital emergency care following a hip fracture is an important outcome and this study highlights the fact that patients expressed a high level of satisfaction with the prehospital emergency care provided by ambulance nurses in both care contexts under study. However, some areas need to be improved in terms of nursing information.

## Background

Prehospital emergency care is an essential part of the healthcare system. Measuring patient satisfaction is of great importance for ensuring that ambulance service care meets expectations and provides the best possible experience. To improve prehospital emergency care and prepare patients for hospitalization, all ambulance organizations in Sweden have upgraded their level of competence by the inclusion of registered nurses since 2015 [1].

Patient dissatisfaction is multidimensional and includes management, quality of health care and the relationship between patients and health care professionals. Moreover, complaints often concern treatment and communication [2].

A study found that patients were dissatisfied with the information provided, organization/rules and perceived that healthcare providers defend themselves when patients complain [3]. Patients also expressed dissatisfaction about waiting times at the A&E for admission to a hospital ward, ineffective communication and lack of environmental control [4].

Dissatisfaction with care is often linked to staff insensitivity and communication failure and healthcare professionals find it challenging to meet patients’ expectations of receiving an explanation and an apology [5]. Other causes of dissatisfaction are lack of knowledge or competence on the part of staff members [6].

Patient satisfaction is a multidimensional concept that measures patients’ experiences of medical competence, including not only clinical and technical skills but also healthcare professionals’ interpersonal skills, attitudes and provision of information [7, 8]. Another dimension of patient satisfaction is associated with patients’ expectations. Patient satisfaction is widely used as a basis for evaluating waiting times and nursing skills in emergency care [9, 10].

Ambulance nurses are qualified to perform assessment, nursing care, medical treatment and information in addition to collaborating with other professionals. Moreover, the National Board of Health and Welfare states that the same standards of diagnosis, treatment and safety should apply in prehospital emergency care as in hospital care [11, 12].

It is well known that older patients suffering from hip fracture require comprehensive care and in Sweden the annual number of cases is predicted to increase from 18,000 today to 30,000 in 2050 [13]. The ambulance service is often the patients’ first care encounter before arriving at the hospital. Progress has been made in prehospital nursing care and many interventions have been transferred from the hospital to the ambulance service [14]. As hip injuries are painful and in most cases the patients are frail, they require specialized emergency care [15]. Registered nurses in ambulance organizations provide care at the scene and during transportation, which includes various forms of assessment, pain treatment, stabilization of the patient’s condition, sending the electrocardiogram (ECG) results to the hospital and providing information to the patient and subsequent caregivers [12]. The prehospital guidelines [16] for patients with suspected hip fracture recommend either the standard procedure with transport to the accident and emergency department (A&E) or prehospital fast track care (PFTC). PFTC means that the ambulance nurse provides a greater number of interventions and prepares the patient for immediate transport to the radiology department and admission to the orthopaedic ward, instead of first transporting her/him to the A&E department.

Several studies describe a lack of satisfaction with ambulance care from the patient perspective. In Finland, dissatisfaction was reported when patients considered that their needs were not met, staff members did not introduce themselves and they did not transport the patients to the hospital they wished to go to [17].

Two studies from the U.S. describe the lack of a professional attitude, rude behaviour, inadequate medical assessment and patient dissatisfaction with the choice of destination [18, 19]. On the other hand, one study from England found that patients experienced ambulance care as very positive [20]. However, the results of studies carried out in Sweden differ and although patients are generally satisfied [21], hip fracture care needs to be improved [22]. Nevertheless, Hommel et al. presented positive statements from patients, which described short waiting times for an ambulance and a fast process on arrival at the hospital and upon admission to an orthopaedic ward [23].

Little is known about patient satisfaction with prehospital emergency care following a hip fracture and whether the ambulance service has succeeded in its mission to strengthen clinical and technical skills as well as sensitive, two-way interpersonal communication with patients.

Greater knowledge in this area will enhance ambulance nurses’ understanding of patients’ expectations, thus providing a valuable basis for guiding knowledge acquisition and competence development in the prehospital area.

The aim of this study was to investigate patients’ satisfaction with prehospital emergency care following a hip fracture by comparing two similar emergency care contexts.

## Methods

### Study design, sample and setting

The patients in this study were recruited from participants in a randomized, controlled trial [24]. The purpose of the original study was to compare two pathways: PFTC and traditional transport to the A&E department, focusing on outcomes in terms of time to radiographic examination and surgery, postoperative complications, length of hospital stay and mortality.

During the study period all patients who were assessed as having a suspected hip fracture by an ambulance nurse were eligible for inclusion. The ambulance nurses informed the patient about the research project and explained the differences in the nursing interventions between the two pathways (PFTC and A&E). If a patient agreed to participate in the study, the randomization took place at the scene by the ambulance nurses using a closed, opaque envelope. In cases where patients were unable to give their consent because of dementia or cognitive deficit, a relative could do so on their behalf. The patients were allocated either to the PFTC (intervention) or to traditional transport to the A&E department (control group).

The inclusion procedure consisted of the ambulance nurse using a study folder marked with the ambulance journal number for each patient who agreed to participate in the study*.*

The present study was designed as a sub-study of the original study, for the purpose of examining patient satisfaction with prehospital emergency care following a hip fracture.

### Prehospital fast track care (PFTC)

The ambulance nurse administered and assessed a 12-Lead ECG and sent it to the hospital database. Blood samples were taken to analyse plasma glucose level. The ambulance nurse provided the patient with an ID-bracelet and called the receptionist or the triage nurse at the A&E department and asked for an x-ray referral to be sent to the radiology department. A phone call was made to the orthopaedic surgeon on duty for confirmation or advice when the ambulance nurse was unsure about the patient’s condition. The orthopaedic ward nurse received information by phone from the ambulance nurse about the patient’s current condition. The patient was transported straight to the radiology department instead of to the A&E department. If the x-ray verified a hip fracture, the patient was transported directly to the orthopaedic ward for preoperative care. If the x-ray did not verify a hip fracture, the patient was transported to the A&E department for further assessment and a decision about treatment.

### Accident and emergency (A&E) department

Patients randomized to A&E were transported to the A&E department and the ambulance nurse reported the patient to the admissions nurse. An A&E nurse gave the patient an ID-bracelet and administered blood tests and an ECG. The patient was placed in an examination room or a corridor along with other orthopaedic patients to await the orthopaedic surgeon. Following examination by the surgeon, the patient was moved to the radiology department for radiographic examination and then back to the A&E department to await the treatment decision. Thereafter the patient was admitted to an orthopaedic department. The A&E nurse then reported the patient to the orthopaedic department and the patient was transported there.

The study was carried out between July 2012 and May 2014 at the ambulance organization in the Region of Halland, Sweden. The organization consists of eight ambulance stations and provides a population of 305,000 people with prehospital emergency care and transport to two emergency hospitals.

### Patients in the study

All patients in the study were assessed by the Rapid Emergency Triage and Treatment System (RETTS) [25] and cared for in accordance with the ambulance organization’s guidelines on pain treatment, oxygen therapy and intravenous liquid substitution.

The inclusion criteria were verified hip fracture**,** awake**,** adequate vision and hearing as well as sufficiently lucid to answer the questionnaire. Patients were excluded if they had other injuries or were affected by dementia (judged by clinical appearance or a known diagnosis) or other conditions that made participation impossible.

### Instrument

The study was conducted using the Consumer Emergency Care Satisfaction Scale (CECSS), which consists of a patient questionnaire that was developed to measure patient satisfaction with A&E nursing care [26]. The instrument was developed in Australia and has been evaluated for validity and reliability when measuring the quality of A&E care from the patient perspective. It contains 19 statements and a 5-point Likert scale is used to measure the patient’s response from *completely agree* = 5 to *completely disagree* = 1.

The CECSS measures patient satisfaction with A&E nursing care in the areas of care and discharge teaching. There are 12 items for care, 3 for discharge teaching and 4 for reducing response bias. The total score ranges from 15 to 75 [27].

A modified version of the instrument was used [21] in which all of the 16 questions measured patient satisfaction, 12 with care (information, clinical and technical skills) as well as 4 negative items (attitude and behaviour) that were summarized separately.

Three items concerning patient teaching were excluded because they measure patient satisfaction before leaving the A&E department and were thus not relevant for this study. The total score was 12–60 for the care subscale and 4–20 for the negative items. A total score of ≥36 on the care subscale indicates patient satisfaction, while < 36 indicates dissatisfaction. For the negative items, ≤12 indicates patient satisfaction and > 12 dissatisfaction.

### Data collection

Patients with a verified hip fracture were admitted to an orthopaedic ward either by means of PFTC or from the A&E department. During the patient’s stay at the orthopaedic ward, a designated nurse from the ambulance service administration, who was not a member of the research team, distributed a modified version of the questionnaire coded with the patient’s ambulance journal number (specific case number, no patient identity). The nurse explained the instructions for filling in the questionnaire pertaining to satisfaction with prehospital emergency care. The patients completed the questionnaire during their hospital stay and returned it to an orthopaedic nurse who stored the questionnaires in the orthopaedic nurses’ office. The designated nurse returned to the orthopaedic ward at a later date and collected the completed questionnaires.

For those patients who did not meet the inclusion criteria and were therefore excluded, the nurse documented the reason for exclusion in the study protocol. Such patients were not provided with information about the questionnaire and their data were not analysed.

Information about age, gender and allocation to either PFTC or A&E was retrieved from the ambulance data.

### Data for comparison with other studies

In order to compare patient satisfaction, the mean score on the care subscale and the number of most positive response alternatives in the present study were compared with the corresponding data from six previous studies conducted between 2002 and 2016 in five different countries [21, 28–32]. The number of patients included varied from 40 to 573. The studies used for comparison were conducted in different emergency care contexts with mixed patient groups and variation in age, gender, ethnicity, priority and nursing interventions.

### Analyses

The outcome data were summarized using descriptive statistics. For comparison between the PFTC and A&E groups, the Mann-Whitney U-Test (with corrections for ties) was employed for ordinal outcomes on individual items and the Chi-square test for the categorized subscale scores. The patients’ sum scores were categorized into 60–48, 47–36 and 35–12 for the care subscale and 4–8, 9–12 and 13–20 for the negative-item subscale.

The analyses were carried out using IBM Statistics for Windows version 20.0.2 [IBM Corp. Armonk, NY, USA].

## Results

### Inclusion and patient characteristics

**Fig. 1:**
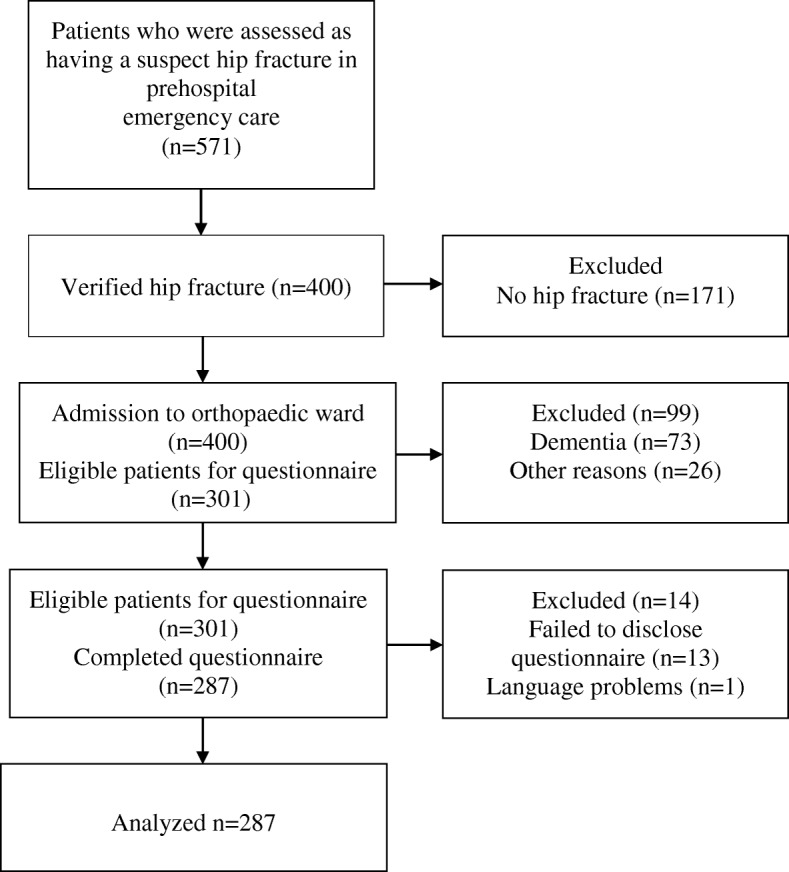
Flow of patients in the questionnaire study

During the study period, the ambulance organization cared for 571 patients with a suspected hip fracture. Of these patients, 284 were excluded (no fracture, *n* = 171; dementia, *n* = 73; other reasons such as declining participation, not remembering being transported or having died during the hospital stay, *n* = 26; failure to complete the questionnaire, *n* = 13; and inadequate knowledge of the Swedish language, n = 1). Hence, 287 patient questionnaires were included and analysed (Fig. 1).

Of the included patients, 188 were women (66%) and 99 were men (34%) with a mean age of 80.9 years. The patient characteristics are presented in Table 1.

**Table 1:** Characteristics of hip fracture patients in prehospital emergency care (*n* = 287) based on the CECSS

Variables of prehospital care	N (%)
PFTC	137 (48)
A&E	150 (52)
Men	99 (34)
Women	188 (66)
Age, mean (years) ± SD^a^	80.9 ± 9.4
Median	83
Min-Max	51–100
ECG	152 (53)
P-glucose	157 (55)
Pain treatment	273 (95)
Oxygen	162 (56)
Infusion	162 (56)
Sedative	81 (28)
Antiemetic	22 (8)

^a^*SD* Standard deviation

### Comparison of the CECSS in PFTC and A&E

There were no significant differences between the PFTC (*n* = 137) and the A&E (*n* = 150) groups in the care subscale; 98% of the patients in both groups indicated satisfaction (*p* = 0.98), with only two from each group reporting dissatisfaction. Considering the negative items, 14% of the patients in the A&E group indicated less satisfaction compared with 6% in the PFTC group (*p* = 0.07) (Table 2). The responses to the two individual negative items, “*The nurse treated me as a* ***‘****case****’*** *instead of as a person”* and *“The nurse was not very friendly”,* differed noticeably between the groups, with a higher proportion of the most favourable response alternatives in the PFTC group (85.4% vs. 80.7%, *p* = 0.21, and 97.1% vs. 85.3%, *p* < 0.01, respectively).

**Table 2:** Comparison of patient satisfaction scores for the care subscale and the negative items

	Score	PFTC (*n* = 137)	A&E (*n* = 150)	*P* value
^a^Care subscale (12 items)	60–48	124	135	0.98^c^
	47–36	11	13	
	35–12	2	2	
^b^Negative items (4 items)	4–8	129	129	0.07^c^
	9–12	6	14	
	13–20	2	7	

^a^ ≥36 indicates patient satisfaction in care items

^b^ ≤ 12 indicates patient satisfaction in negative items

^c^ Chi-2 test was used for comparison of the category scores

### Patient satisfaction – Distribution of responses

The distribution of responses on patient satisfaction for each item on the care subscale and negative item scale is presented in Table 3 with the number and percentage for each response. More than 80% of the patients selected the most positive response alternative on both the care subscale and the negative questions. However, two items on the care subscale concerning nursing information, namely *“The nurse gave me a chance to ask questions”* and *“The nurse made sure that all my questions were answered”,* revealed that 16.4 and 16.7% respectively of the patients were dissatisfied. Mean scores for the care subscale and the negative items were 55.47 (SD 5.7) and 5.52 (SD 2.8) respectively.

**Table 3:** Number and percentage distribution of CECSS item responses (*n* = 287)

Item	N (%)				
	Total agreement		Total disagreement		
	5	4	3	2	1
1.The nurse performed her/his duties with skill	266(92.4)	16(5.6)	4 (1.4)	0 (0.0)	1 (0.3)
2.The nurse seemed to know something about my illness/problem	263 (91.6)	18 (6.3)	5 (1.7)	1 (0.3)	0 (0.0)
3.The nurse knew what treatment I needed	246 (85.7)	19 (6.6)	16(5.6)	3 (1.0)	3 (1.0)
4.The nurse should have been more attentive than he/she was	21 (7.3)	6 (2.1)	3 (1.0)	16(5.6)	241 (84.0)
5.The nurse explained all procedures before they were carried out	225 (78.4)	26 (9.1)	19 (6.6)	10 (3.5)	7 (2.4)
6.The nurse seemed too busy to spend time talking to me	16(5.6)	3 (1.0)	7 (2.4)	13 (4.5)	248 (86.4)
7.The nurse explained things in terms I could understand	246 (85.7)	17 (5.9)	14 (4.9)	7 (2.4)	3 (1.0)
8.The nurse was understanding when listening to my problem	230 (80.1)	20 (7.0)	19 (6.6)	12 (4.2)	6 (2.1)
9.The nurse seemed genuinely concerned about my pain, fear and anxiety	237 (82.6)	22 (7.7)	15 (5.2)	7 (2.4)	6 (2.1)
10.The nurse was as gentle as he/she could be when performing painful procedures	258 (89.9)	16(5.6)	9 (3.1)	2 (0.7)	2 (0.7)
11.The nurse treated me as a “case” instead of as a person	23 (8.0)	4 (1.4)	8 (2.8)	14 (4.9)	238 (82.9)
12.The nurse seemed to understand how I felt	239 (83.3)	26 (9.1)	15 (5.2)	5 (1.7)	2 (0.7)
13.The nurse gave me a chance to ask questions	172 (59.9)	25 (8.7)	43 (15.0)	24 (8.4)	23 (8.0)
14.The nurse was not very friendly	14 (4.9)	3 (1.0)	3 (1.0)	6 (2.1)	261 (90.9)
15.The nurse appeared to take time to meet my needs	239 (83.3)	27 (9.4)	11 (3.8)	7 (2.4)	3 (1.0)
16 The nurse made sure that all my questions were answered	174 (60.6)	24 (8.4)	41 (14.3)	27 (9.4)	21 (7.3)
Care subscale (12 items)	233 (81.2)	21 (7.3)	18 (6.2)	9 (3.1)	6 (2.0)
Negative item subscale (4 items)	19 (6.6)	4 (1.4)	5 (1.7)	12 (3.9)	247 (86)

### Comparison with other studies using the CECSS

Four out of six studies reported a high level of patient satisfaction. In five of the studies, the mean scores on the care subscale ranged from 43.46 to 57.60. One study reported the results as percentages of the most positive response alternatives. Two studies reported a higher level of patient satisfaction compared with the present study, one with a mean score on the care subscale and the other with the most positive response alternatives (Table 4).

**Table 4:** Studies employing the CECSS for comparison with the present study

Author	Year	Number of patients	Country	Result	Score or most positive response alternatives
Cunado et al. [28]	2002	96	Spain	High satisfaction	50.50^a^
Chan JN, Chau J. [29]	2005	56	Hong Kong	Satisfaction	43.93^a^
Ekwall A, Davies BA. [30]	2010	157	Sweden	High satisfaction	45.9–52.6^a^
Johansson et al. [21]	2011	40	Sweden	High satisfaction	93% most positive response alternatives^b^
Wright et al. [31]	2013	573	USA	High satisfaction	55.9–57.6^a^
Messina et al. [32]	2014	259	Italy	Satisfaction	43.46^a^
Larsson et al.^c^	2018	287	Sweden	High satisfaction	55.47^a^ 82.5% most positive response alternatives^b^

^a^ Mean score on the care subscale

^b^ Proportion of the most positive response alternatives

^c^ The present study

## Discussion

This study demonstrates that patients with a hip fracture were satisfied with the care provided by registered nurses during ambulance assignments. A majority of the patients selected the most positive response alternative on the care subscale and also in response to the negative questions. Some responses, especially on the care subscale regarding “skills, knowledge and concern”, indicate a very high level of patient satisfaction with prehospital emergency care.

No significant difference was observed between the PFTC and the A&E groups on the care subscale. A streamlined process with faster admission to a hospital had no effect on patient satisfaction in our study, although there was a difference in terms of satisfaction with the greater number of nursing interventions associated with PFTC. The waiting time and competence associated with fast track care at A&E have previously been reported to be a predictor of patient satisfaction [33]. However, there was a tendency towards less patient satisfaction in the A&E group compared with the PFTC group on the negative items. Patients in the PFTC group responded more positively on specific aspects of nurses’ caring behaviour. The importance for patient satisfaction might be on negative items, possibly highlighting the importance that the patient attributes to the fact that the ambulance nurse facilitated faster admission on arrival at the hospital. Accordingly, this study suggests a possible area in which patient satisfaction could be improved.

Patients with a hip fracture often suffer severe pain and anxiety. Ambulance nurses provide immediate care at the scene comprising pain relief, examination, removal and transport to hospital. It is reasonable to assume that the positive response largely depends on the rapid aid as well as the competence and carefulness of the ambulance nurses, which together result in patients feeling better, due to a reduction in their pain and anxiety. A recently published study describes positive experiences when ambulance personnel used different pain management strategies for patients with a suspected hip fracture [34].

“The nurse seemed genuinely concerned about my pain, fear and anxiety” is one example of a statement that received a high level of the most positive response alternatives, which definitely underlines the high quality of care provided by ambulance nurses. One previous study describes patients’ positive experiences of prehospital emergency care, but also certain negative effects of medical treatment**,** such as confusion and the need to ask questions about what really happened in the ambulance [22].

Dissatisfaction with care is often related to lack of information and communication [6].

The present study reveals a positive response to questions dealing with these areas**,** which may be due to the development of prehospital guidelines and awareness of the importance of high-quality care for this vulnerable group of patients.

Despite the acute situation, the ambulance nurse has time to talk and listen to the patient. Informing a patient about what is going to happen and reporting the patient’s condition to the next level of care ensure a continuum of care. However, 16% of patients were dissatisfied with the nursing information received. Despite the generally very positive responses from patients, this is an important finding and indicates areas that require improvement. Another prehospital study describes the patients’ deep need for appropriate information to enhance their experience. Relational skills together with technical knowledge contributed to patients’ perception of professionalism in the ambulance service [35]. Accordingly, an increased focus on physical, emotional and social needs might contribute to greater patient satisfaction.

In addition, communicational and behavioural skills have previously been described as important for ambulance nurses’ competence [36].

### Comparison with other studies

Despite the fact that The National Board of Health and Welfare has stated that prehospital emergency care standards should be identical to those in hospital emergency care, there is a gap in the literature concerning patient satisfaction with nursing care in the ambulance service. We therefore compared patient satisfaction from two similar emergency care contexts using the same questionnaire.

The CECSS has been used in several studies and different emergency care contexts. While we are aware that the modified version for ambulance care has not been tested for reliability and validity, we believe the two contexts to be comparable.

In comparison with other studies, our result indicates a high level of patient satisfaction.

Only one other study presents a higher mean score on the care subscale than the present study [31]. However, the score level of all the studies indicates satisfaction or a very high level of satisfaction [28–30, 32]. When compared with a previous study from Sweden on the ambulance service, the present study indicates a very high level of patient satisfaction [21]. In addition, comparable studies describe patient satisfaction in different and unspecified patient groups. Another aspect that should be considered is that the present study focused on a large and specific patient group requiring skilled nursing care, thus contributing reliable information about satisfaction with prehospital emergency care in patients with a hip fracture.

### Strengths and limitations

The response rate was high, indicating a great willingness to participate in the study.

It is reasonable to assume that the result can be generalized to other ambulance organizations in which ambulance nurses provide care and use similar guidelines for this patient group.

Some considerations should be borne in mind. For example, patients with dementia were excluded. One solution might have been for relatives to answer the questionnaire. A previous study using the CECSS describes options for the participation of accompanying persons [37].

As one cannot rely on relatives being present at the scene, either in the ambulance or at the hospital, we decided not to use the option of proxy answers for individuals with dementia.

Investigating patient satisfaction and gaining knowledge of how to achieve quality improvements in healthcare are recognised as challenging [38]. Studies using questionnaires are relatively simple to implement and constitute an approved method for investigating quality of care. However, as several studies have concluded that all participants were more or less satisfied, it is possible that patient satisfaction is far too general a parameter to be examined by means of a questionnaire and that the instrument is not specific enough about what patients are satisfied or dissatisfied with. It is reasonable to assume that patients’ expectations of ambulance care vary between individuals, depending on morbidity and other factors that may affect individual patient satisfaction. In order to increase knowledge about patient satisfaction with prehospital emergency care, it will be necessary to develop new methods, probably with a more individual approach, such as phone calls, e-mails or deep interviews, where patients themselves can decide and explain what they are satisfied or dissatisfied with.

Although the quality of ambulance care is often defined by waiting times for life-threatening conditions, some authors have addressed the need for quality indicators in prehospital emergency care [39]. Patients may have low expectations of prehospital emergency care, knowing little about it in its modern form. They assume they will just be given basic assistance at the scene and then transportation, not the more advanced types of care provided today [40]. This might explain the high level of satisfaction in the present study.

In other words, patients’ expectations are met if they feel that they receive adequate physical care and encounter a friendly attitude [14]. Although it is challenging to investigate patient satisfaction, the results of the present study indicate certain areas that require further research. Firstly, patient expectations must be addressed individually and in detail to understand the background and reasons for individual patient satisfaction. No two patients are likely to be identical in this respect. Secondly, improving the nursing information given to patients is essential, as is the actual delivery of information in an effective and authoritative manner. Thirdly, evaluations of patient expectations and satisfaction should be undertaken on a regular, systematic basis in order to guide the development of competence in the ambulance service. Fourthly, more prehospital emergency care outcomes need to be documented from the patient perspective, leading to the establishment of a set of quality indicators. Other authors have also described the need for quality indicators in prehospital emergency care [41]. These four indications thus point conclusively to the need for further research on several aspects of the topic investigated in this study.

## Conclusion

It is essential to examine patient satisfaction with prehospital emergency care following a hip fracture. This study highlights patients’ high level of satisfaction with the prehospital emergency care provided by ambulance nurses. The ambulance service has succeeded in its mission to develop and strengthen prehospital emergency skills in the care of patients with a hip fracture. However, several areas can be improved in terms of nursing information, regular evaluations and the establishment of a set of quality indicators for prehospital emergency care.

## Abbreviations

A&E: Accident and emergency department
CECSS: Consumer Emergency Care Satisfaction Scale
ECG: Electrocardiogram
PFTC: Prehospital fast track care
RETTS: Rapid Emergency Triage and Treatment System

## References

[1] Sjolin H, Lindstrom V, Hult H, Ringsted C, Kurland L (2015). What an ambulance nurse needs to know: a content analysis of curricula in the specialist nursing programme in prehospital emergency care. Int Emerg Nurs.

[2] Reader TW, Gillespie A, Roberts J (2014). Patient complaints in healthcare systems: a systematic review and coding taxonomy. BMJ Qual Saf.

[3] Skalen C, Nordgren L, Annerback EM (2016). Patient complaints about health care in a Swedish County: characteristics and satisfaction after handling. Nurs Open.

[4] Lee AV, Moriarty JP, Borgstrom C, Horwitz LI (2010). What can we learn from patient dissatisfaction? An analysis of dissatisfying events at an academic medical center. J Hosp Med.

[5] Cave J, Dacre J (2008). Dealing with complaints. BMJ.

[6] Sverige. Socialstyrelsen (2009). Nationella indikatorer för god vård: hälso- och sjukvårdsövergripande indikatorer: indikatorer i Socialstyrelsens nationella riktlinjer.

[7] Dinh M, Walker A, Parameswaran A, Enright N (2012). Evaluating the quality of care delivered by an emergency department fast track unit with both nurse practitioners and doctors. Australia Emerg Nurs J.

[8] Taylor C, Benger JR (2004). Patient satisfaction in emergency medicine. Emerg Med J.

[9] Jennings N, Clifford S, Fox AR, O'Connell J, Gardner G (2015). The impact of nurse practitioner services on cost, quality of care, satisfaction and waiting times in the emergency department: a systematic review. Int J Nurs Stud.

[10] Shital S, Anay K, Rumoro Dino P, Samuel HF (2015). Managing patient expectations at emergency department triage. Patient Exp J.

[11] Suserud B-O (2005). A new profession in the pre-hospital care field - the ambulance nurse. Nurs Crit Care.

[12] Socialstyrelsen. SOSFS 2009:10 (2009). Socialstyrelsens föreskrifter om ambulanssjukvård m.m.

[13] Rosengren BE, Karlsson MK (2014). The annual number of hip fractures in Sweden will double from year 2002 to 2050: projections based on local and nationwide data. Acta Orthop.

[14] Melby V, Ryan A (2005). Caring for older people in prehospital emergency care: can nurses make a difference?. J Clin Nurs.

[15] Hwang U, Richardson LD, Morrison RS, Sonuyi TO (2006). The effect of emergency department crowding on the management of pain in older adults with hip fracture. J Am Geriatr Soc.

[16] Suserud B, Lundberg L (2016). Prehospital emergency care. 2, [revised and extended] ed.

[17] Kuisma M, Maatta T, Hakala T, Sivula T, Nousila-Wiik M (2003). Customer satisfaction measurement in emergency medical services. Acad Emerg Med.

[18] Colwell CB, Pons PT, Pi R (2003). Complaints against an EMS system. J Emerg Med.

[19] Doering GT (1998). Customer care. Patient satisfaction in the prehospital setting. Emerg Med Serv.

[20] Halter M, Marlow T, Tye C, Ellison GT (2006). Patients’ experiences of care provided by emergency care practitioners and traditional ambulance practitioners: a survey from the London ambulance service. Emerg Med J.

[21] Johansson A, Ekwall A, Wihlborg J (2011). Patient satisfaction with ambulance care services: survey from two districts in southern Sweden. Int Emerg Nurs.

[22] Aronsson K, Bjorkdahl I, Wireklint SB (2014). Prehospital emergency care for patients with suspected hip fractures after falling - older patients’ experiences. J Clin Nurs.

[23] Hommel A, Kock ML, Persson J, Werntoft E (2012). The Patient's view of nursing care after hip fracture. ISRN Nurs.

[24] Larsson G, Stromberg RU, Rogmark C, Nilsdotter A (2016). Prehospital fast track care for patients with hip fracture: impact on time to surgery, hospital stay, post-operative complications and mortality a randomised, controlled trial. Injury.

[25] Widgren BR, Jourak M (2011). Medical emergency triage and treatment system (METTS): a new protocol in primary triage and secondary priority decision in emergency medicine. J Emerg Med.

[26] Davis BA, Bush HA (1995). Developing effective measurement tools: a case study of the consumer emergency care satisfaction scale. J Nurs Care Qual.

[27] Davis BA, Kiesel CK, McFarland J, Collard A, Coston K, Keeton A (2005). Evaluating instruments for quality: testing convergent validity of the consumer emergency care satisfaction scale. J Nurs Care Qual.

[28] Cunado BA, Garcia CB, Rial CC, Garcia LF (2002). Spanish validation of an instrument to measure the quality of nursing care in hospital emergency units. J Nurs Care Qual.

[29] Chan JN, Chau J (2005). Patient satisfaction with triage nursing care in Hong Kong. J Adv Nurs.

[30] Ekwall A, Davis BA (2010). Testing a Swedish version of the consumer emergency care satisfaction scale in an emergency department and 2 observation wards. J Nurs Care Qual.

[31] Wright G, Causey S, Dienemann J, Guiton P, Coleman FS, Nussbaum M (2013). Patient satisfaction with nursing care in an urban and suburban emergency department. J Nurs Adm.

[32] Messina G, Vencia F, Mecheroni S, Dionisi S, Baragatti L, Nante N (2014). Factors affecting patient satisfaction with emergency department care: an Italian rural hospital. Glob J Health Sci.

[33] Dinh MM, Enright N, Walker A, Parameswaran A, Chu M (2013). Determinants of patient satisfaction in an Australian emergency department fast-track setting. Emerg Med J.

[34] Jakopovic D, Falk AC, Lindstrom V (2015). Ambulance personnel's experience of pain management for patients with a suspected hip fracture: a qualitative study. Int Emerg Nurs.

[35] Togher FJ, Davy Z, Siriwardena AN (2013). Patients’ and ambulance service clinicians’ experiences of prehospital care for acute myocardial infarction and stroke: a qualitative study. Emerg Med J.

[36] Wihlborg J, Edgren G, Johansson A, Sivberg B (2017). Reflective and collaborative skills enhances ambulance nurses’ competence - a study based on qualitative analysis of professional experiences. Int Emerg Nurs.

[37] Kristensson J, Ekwall A (2008). Psychometric properties of the consumer emergency care satisfaction scale: tested on persons accompanying patients in emergency department. J Nurs Care Qual.

[38] Al-Abri R, Al-Balushi A (2014). Patient satisfaction survey as a tool towards quality improvement. Oman Med J.

[39] Price L (2006). Treating the clock and not the patient: ambulance response times and risk. Qual Saf Health Care.

[40] Ahl C, Nystrom M, Jansson L (2006). Making up one’s mind:--patients’ experiences of calling an ambulance. Accid Emerg Nurs.

[41] Pittet V, Burnand B, Yersin B, Carron PN (2014). Trends of pre-hospital emergency medical services activity over 10 years: a population-based registry analysis. BMC Health Serv Res.

